# Disease Progression and Mutation Pattern in a Large Cohort of LGMD R1/LGMD 2A Patients from India

**DOI:** 10.1055/s-0041-1736567

**Published:** 2021-11-09

**Authors:** Valakunja H. Ganaraja, Kiran Polavarapu, Mainak Bardhan, Veeramani Preethish-Kumar, Shingavi Leena, Ram M. Anjanappa, Seena Vengalil, Saraswati Nashi, Gautham Arunachal, Swetha Gunasekaran, Dhaarini Mohan, Sanita Raju, Gopikrishnan Unnikrishnan, Akshata Huddar, Valasani Ravi-Kiran, Priya T. Thomas, Atchayaram Nalini

**Affiliations:** 1 Department of Neurology, National Institute of Mental Health and Neurosciences, Bengaluru, Karnataka, India; 2 Division of Neurology, Department of Medicine, Children's Hospital of Eastern Ontario Research Institute, University of Ottawa, The Ottawa Hospital, Ottawa, Canada; 3 Department of Human Genetics, National Institute of Mental Health and Neurosciences, Bengaluru, Karnataka, India; 4 Department of Psychiatric Social Work, National Institute of Mental Health and Neurosciences, Bengaluru, Karnataka, India

**Keywords:** *CAPN3*, muscular dystrophy, LGMDR1, next-generation sequencing

## Abstract

Calpainopathy is caused by mutations in the *CAPN3* . There is only one clinical and genetic study of *CAPN3* from India and none from South India. A total of 72 (male[M]:female [F] = 34:38) genetically confirmed probands from 72 independent families are included in this study. Consanguinity was present in 54.2%. The mean age of onset and duration of symptoms are 13.5 ± 6.4 and 6.3 ± 4.7 years, respectively. Positive family history occurred in 23.3%. The predominant initial symptoms were proximal lower limb weakness (52.1%) and toe walking (20.5%). At presentation, 97.2% had hip girdle weakness, 69.4% had scapular winging, and 58.3% had contractures. Follow-up was available in 76.4%, and 92.7% were ambulant at a mean age of 23.7 ± 7.6 years and duration of 4.5 years, remaining 7.3% became wheelchair-bound at 25.5 ± 5.7 years of age (mean duration = 13.5 ± 4.6), 4.1% were aged more than 40 years (duration range = 5–20). The majority remained ambulant 10 years after disease onset. Next-generation sequencing (NGS) detected 47 unique *CAPN3* variants in 72 patients, out of which 19 are novel. Missense variants were most common occurring in 59.7% (homozygous = 29; Compound heterozygous = 14). In the remaining 29 patients (40.3%), at least one suspected loss of function variant was present. Common recurrent variants were c.2051–1G > T and c.2338G > C in 9.7%, c.1343G > A, c.802–9G > A, and c.1319G > A in 6.9% and c.1963delC in 5.5% of population. Large deletions were observed in 4.2%. Exon 10 mutations accounted for 12 patients (16.7%). Our study highlights the efficiency of NGS technology in screening and molecular diagnosis of limb-girdle muscular dystrophy with recessive form (LGMDR1) patients in India.

## Introduction

Calpainopathy caused by mutations in the *CAPN3* gene (OMIM:114240) is one of the most common types of autosomal recessive limb-girdle muscular dystrophy (ARLGMD). The disease onset ranges from early childhood to adulthood and is typically characterized by progressive symmetrical weakness and wasting of proximal limb muscles and contractures. 1 While the recessive form of LGMD (LGMDR1) is the most common one, the autosomal dominant form (LGMDD4) of calpainopathy has been reported in few families with adult onset and milder phenotypes. 2 3 *CAPN3* gene encodes a protease called calpain3 and is located in the chromosome region 15q15.1–q21.1. 4 *CAPN3* is a Ca ^2+^ dependent protein comprising of 821 amino acids. 5 6 The gene consists of 24 exons and produces a skeletal muscle-specific homodimer cysteine protease belonging to the calpain superfamily which is involved in myofibrillogenesis and sarcomere remodeling. 7 8 9 10 The prevalence of calpainopathy among all dominant and recessive forms of LGMD's ranges from 11% in the United States and Mexico, 11 12 13 to 40 to 50% in Turkey, India, and Bulgaria, 14 15 16 17 and even higher in Basque and Russia population. 18 19 20

Although a few reports based on western blot confirmation of calpainopathy are published on Indian patients, 21 22 only two studies describe the mutation pattern in the Indian community with calpainopathy and none on the disease progression. 23 24 Additionally, a few international studies from Italy and France reported on the natural history/disease progression. 25 26 27

In the current study, we elaborated on the clinical presentation, disease progression, and mutation pattern of a large cohort of genetically confirmed LGMDR1 patients evaluated at a single quaternary referral center for neurological disorders in India. Since we will be discussing only the recessive form, the term “LGMDR1” will be used synonymously with “calpainopathy.”

## Materials and Methods

Institutional ethics committee approval for the study was obtained (NIMHANS/IEC/2020–21). This is a retrospective analysis with a description of the clinical phenotype and mutation pattern in 72 patients of genetically confirmed LGMDR1 patients. They belonged to a cohort of 345 ARLGMD patients evaluated between 2016 and 2020 at the multidisciplinary neuromuscular disorders clinic. After detailed history taking and neurological examination of all patients, relevant demographic and clinical data were entered in SPSS (Statistical Package for the Social Sciences) for further statistical analysis. It is commonly used for analysis of complex statistical data. The muscle strength was evaluated by the modified Medical Research Council (MRC) grading scale 28 which is an objective method used clinically to assess individual muscle weakness. After obtaining written informed consent, patients underwent genetic testing by next-generation sequencing (NGS) including clinical exome or whole exome sequencing. The libraries were sequenced on the Illumina sequencing platform, followed by bioinformatic analysis customized for calling single nucleotide variants (SNVs), small insertion/deletions (INDELs), as well as larger copy number variants (CNVs; exon deletions and duplications). *CAPN* 3 variant annotation was done based on GenBank transcript: NM_000070.3. We used the free version of VarSome online tool ( *https://www.varsome.com/* ) and manual application of American College of Medical Genetics and Genomics (ACMG) criteria (2015) 29 for classification of SNVs and INDELs. CNVs when identified were automatically considered as causing loss of function and classified as pathogenic. Multiplex ligation-dependent probe amplification (MLPA) was also performed in two patients with large multiexon deletions identified by NGS.

We checked available data in Clinvar, Human Gene Mutation Database (HGMD), Leiden variant database ( *https://databases.lovd.nl/shared/genes/CAPN3* ), and literature for identification of reported variants. Demographic and phenotype data of patients were entered in SPSS Version 23 for statistical analysis. To study the disease progression with respect to time to loss of independent ambulation and time to wheel chair and bed bound state, all the details were obtained from the medical records. A simple questionnaire with information for functional and physical disability was posted to all genetically confirmed LGMDR1 patients to collect their latest disability status.

## Statistics

Data were analyzed in SPSS version 23 using descriptive statistics, such as mean and standard deviation for continuous variables. Frequency percentage was used for categorical variables. Comparison between two genotype groups was performed by independent sample *t* -test. The significance level ( *p* -value) was fixed at 0.05.

## Results

Among the 345 genetically confirmed ARLGMD patients, we identified 72 LGMDR1 (20.9%) individuals from 72 independent families. The mean age at onset was 13.5 ± 6.4 (range: 1–35) years, and the mean duration was 6.3 ± 4.7 (range: 0.4–20) years. There were 34 (47.2%) males. Patients from the southern states of India formed 75.4% of the study cohort, followed by 17.8% from the Eastern states, while the remaining (6.9%) were from the West and Northern parts. Consanguinity was reported in 39 (54.2%) patients, and a family history of similar illness was present in 17 (23.3%). About 29 (40.3%) patients had onset before 12 years of age, while 35 (48.6%) had onset between 12 and 20 years of age, and only 8 (11.1%) had onset after 20 years. The initial symptoms were progressive proximal lower limb weakness (52.1%), toe walking (20.5%), and difficulty running fast (9.6%), while a small proportion (5.5%) had a combination of these at the onset. The clinical features are summarized in Table 1 .

**Table 1:** Clinical profile of 72 patients of calpainopathy

Parameter	Total number( *n* = 72)	Percentage
Duration of illness (y)	6.3 ± 4.7	
Male:female	34:38	
Consanguinity	39	54.2
Family history	17	23.3
Initial symptom		
Age of onset (y)	13.5 ± 6.4	
Proximal lower limb weakness	38	52.1
Toe walking	15	20.5
Difficulty in running fast	7	9.6
Combination of symptoms	4	5.5
Repeated falls	4	5.5
Muscle pain	2	2.8
Calf swelling	1	1.4
Delayed motor milestones	1	1.4
Symptoms at presentation		
Difficulty in climbing stairs	70	97.2
Difficulty in getting up	70	97.2
Proximal upper limb weakness	48	66.6
Running difficulty	41	56.9
Recurrent falls	36	50
Muscle pain	12	16.6
Exercise intolerance	9	12.5
Distal upper limb weakness	6	8.3
Distal lower limb weakness	2	2.7
Examination		
Scapular winging	50	69.4
Contractures	42	58.3
Toe walking	29	40.2
Lumbar lordosis	28	38.8
Calf hypertrophy	24	33.3

At presentation, 70 (97.2%) patients had difficulty in rising from the floor and climbing stairs from mean ages of 14.5 ± 6.1 and 14.9 ± 5.8 years, respectively. Recurrent falls were reported by 36 (50%) patients from a mean age of 15.6 ± 6.0 years. About 48 (66.6%) patients presented with proximal upper limb weakness from 16.7 ± 5.7 years of age, and a small proportion of 6 (8.3%) patients had features of distal upper limb weakness with later onset at the age of 17.7 ± 4.8 years. About 41 (56.9%) patients had observed that they had difficulty in running like others from a mean age of 14.7 ± 6.8 years. Other presentations were muscle pain and exercise intolerance in 12 (16.6%) and 9 (12.5%) patients, respectively. Two (2.7%) were in wheelchair/bedbound state at 18 and 26 years of age with 11 and 14 years into their illness, respectively. None of the patients had symptoms to suggest cranial muscle involvement.

**Fig. 1:**
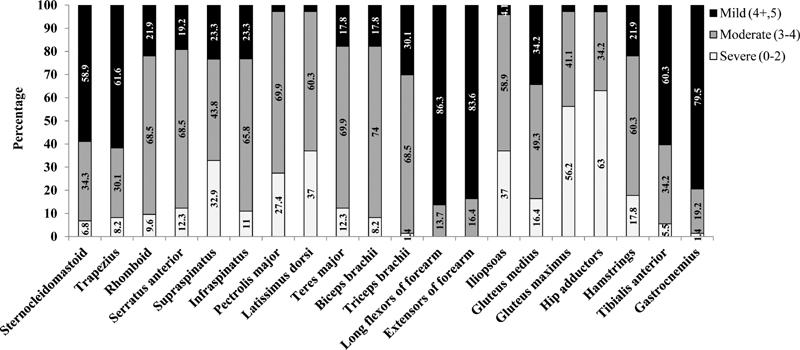
Severity of various muscle weakness among 72 patients with “calpainopathy.”

On examination, approximately 28 (38.8%) had prominent lumbar lordosis, 24 (33.3%) had mild calf hypertrophy, and 13 (18.1%) patients had prominent atrophy of calves and arms. Scapular winging was noticed in 50 (69.4%) and prominent contractures in 42 (58.3%) patients, predominantly at the ankles and knees. Around 15 (20.8%) patients had mild facial weakness. The muscle strength was severely reduced in the girdle muscles ( Fig. 1 ). Preferential involvement of the muscles was recorded: pectoralis and biceps muscles were more severely involved than deltoids and triceps. Iliopsoas, hip adductors, and gastrocnemius were more affected than the glutei and anterior leg muscles. The pattern of muscle involvement is summarized in Table 2 . Distal muscles were preserved in the majority of patients ( Fig. 1 ). All individuals had a waddling gait associated with prominent toe walking in 29 (40.3%). Tendon reflexes were generally hypoactive but ankle jerk was preserved in 52 (72.2%) patients.

**Table 2:** Muscle selectivity pattern in calpainopathy patients

Selectivity pattern	Number	Percentage
Upper limbs		
Deltoid > pectoralis	8	10.9
Deltoid = pectoralis	25	35.6
Deltoid < pectoralis	39	53.4
Arm		
Biceps > triceps	36	50.6
Biceps = triceps	29	39.7
Biceps < triceps	7	9.5
Lower limbs		
Iliopsoas > gluteus maximus	15	20.5
Iliopsoas = gluteus maximus	26	35.6
Iliopsoas < gluteus maximus	31	43.8
Thigh		
Adductors > abductors	49	67.1
Adductors = abductors	19	27.4
Adductors < abductors	4	5.5
Leg		
Gastrocnemius > tib anterior	4	5.5
Gastrocnemius = tib anterior	51	69.9
Gastrocnemius < tib anterior	17	24.7

### Genetic Findings

**Fig. 2:**
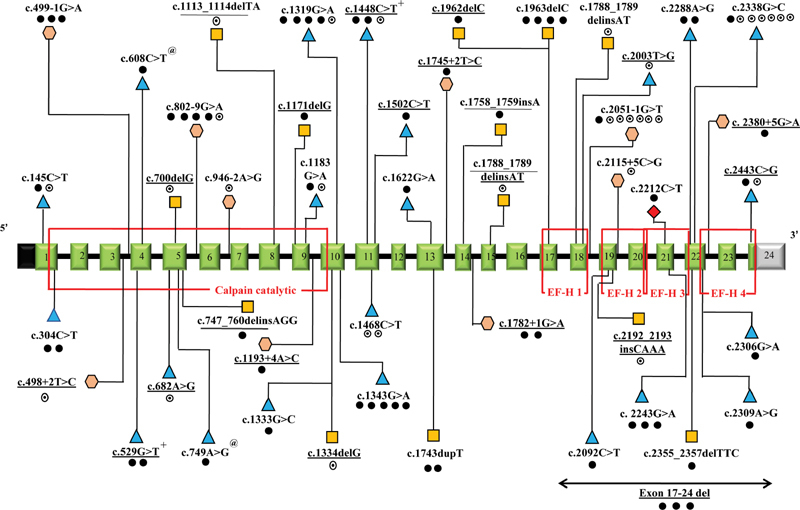
Schematic representation of the variations identified in our study in *CAPN3* gene with corresponding exons and protein domains. The 24 exons of *CAPN3* (NM_000070.3) are represented as boxes with respective exonic numbers with noncoding regions shaded in black and gray at the ends. The domain information is based on UniProtKB - P20807 (CAN3_HUMAN) taken from Uniprot database ( *https://www.uniprot.org/* ). The novel variants are underlined. The dark dots represent homozygous variations, unfilled dots represent heterozygous variations, semifilled dot represent compound heterozygous variations. Triangles represent missense, squares represent frameshift, rhombus represent nonsense and hexagon represent intronic variations. ; @, +, - Individuals carry two homozygous variations in *CAPN3* . The size of exons /introns is not represented at scale. Calpain catalytic domain and EF-hand domains 1–4 are shown in red.

Among the 72 genetically confirmed LGMDR1 patients, 57 had homozygous and the remaining 15 had compound heterozygous mutations in the *CAPN3* gene. Among the compound heterozygous cases, all except two had missense variant in one allele with suspected loss of function variant like frameshift causing INDEL or splice affecting intronic substitution in the opposite allele. Two compound heterozygous patients had biallelic INDELs and missense variants, respectively (Pt_20 and Pt_28). Out of 57 homozygous cases, 29 had missense variants, 28 had suspected loss of function variants (splice affecting intronic variants = 13; INDELs causing frameshift = 10; multiexonic deletions = 3; inframe 3 bp deletion = 1; and substitution resulting in nonsense codon = 1). In total, 47 unique disease-causing *CAPN3* variants were identified in this study, out of which 19 are novel mutations ( Fig. 2 ; Supplementary Table S1 , available in online version only).

While disease causing variants are scattered across the *CAPN3* gene with no specific hotspot regions, we identified few recurrent variants in Indian patients ( Table 4 ). The commonest mutation identified are c.2051–1G > T and c.2338G > C in seven (9.7%) patients each. Other mutation clusters were c.802–9G > A, c.1319G > A, and c.1343G > A occurring in five (6.9%) patients each, while c.1963delC was identified in four (5.5%) patients. Exon 10 was most frequently involved in our patients with 12 mutations (16.7%). No mutations were observed in exons 2, 3, 7, and 12. Large deletions were observed between exons 17 to 24 in three unrelated patients by NGS. We confirmed the deletion in two of the three patients with additional deletion/duplication analysis by MLPA. The family members of 31 patients were screened by segregation analysis and at least one member was found to be having a heterozygous state, and six members with similar illness were detected to have homozygous status ( Supplementary Table S2 , available in online version only).

### Genotypic Patterns and Disease Progression

Clinical follow-up details were available in 55 (76.4%) patients with a mean duration of 4.5 ± 4.3 years. About 92.7% of them were ambulant at a mean age of 23.7 ± 7.6 years, while the remaining 7.3% (four patients) had become wheelchair bound at a mean age of 25.5 ± 5.7 years, after 13.5 (13.5 ± 4.6) years into the illness. On subgroup analysis among patients with different mutations, those with missense mutations had later age of presentation and slow disease progression than those with other mutations ( Table 3 ). Among patients in wheelchair-bound state, only one had missense mutation (Pt_64). Three patients (4.1%) were available for follow-up with age more than 40 years and disease duration ranging from 5 to 20 years (Pt_26, Pt_36, and Pt_39); all were ambulant with assistance of which two were having missense mutations. The oldest patient with follow-up in our study cohort is 43 years, with 20 years of illness duration (Pt_26). One patient (Pt_17) with splice affecting mutation (c.802–9G > A) had sudden death at 21 years of age after 7 years into the illness and was reported to be due to cardiac arrest; however, medical records are not available. None of the patients had features suggestive of respiratory distress during the period of clinical follow-up.

**Table 3:** Genotypic correlation among calpainopathy patients with clinical follow-up ( *n* = 55)

Parameters	Group 1: *n* = 24 (missense mutation)	Group 2: *n* = 31 (other mutations)	*p* -Value
Age of onset (y)	16.4 ± 7.0	11.7 ± 5.66	0.07
Duration (y)	6.4 ± 4.4	6.4 ± 5.3	0.9
Age at presentation (y)	22.8 ± 7.1	18.13 ± 7.0	0.02
Male:female	12:12	12:19	NA
Consanguinity (yes:no)	14:10	17:14	NA
Presence of family history	8	5	NA
Mean age of follow-up (y)	25.4 ± 7.5	22.2 ± 8.3	0.14
Wheelchair bound status during follow-up	1	3	NA

Abbreviation: NA, not applicable.

## Discussion

In the current study, we present the results of clinical findings, pattern of disease progression, and genetic mutation profile of a large number of LGMDR1 patients from India. The majority of them belong to southern India and demonstrated varied mutation patterns. In an earlier study from India, LGMDR1 is reported to be the commonest form of ARLGMD; however, there is no clear data about the exact prevalence and subtypes of genetically confirmed patients. 14 In our study, LGMDR1 constituted 21.2% of all genetically confirmed ARLGMD patients.

The age of onset of muscle weakness in the present study is in concordance with previous studies 20 22 ; however, a later age of onset has been noted in a study from Japan. 30 Both genders were equally affected in our study, similar to the findings in other large series. 31 32 However, in one study from India with 75 patients, male predominance was noted. 14

Nearly half of our patients had proximal weakness of lower limbs as the initial presentation and almost 20% had tip-toe walking. Scapular winging was noted in 69% of patients. The commonest presentation described in other studies are similar with difficulty in running, tip-toe walking, and scapular winging along with proximal lower limb weakness. 25 32 33 In a study by Peric et al, from Serbia, up to 74% of their patients had features of proximal lower limb weakness as the initial presentation, and scapular winging was noticed in more than two-thirds of their patients. 34

The pectorals, biceps brachii, and gluteus maximus were the weakest muscles. This pattern of selective involvement is also reported in a previous study. 35 The symmetrical and preferential muscle involvement also helps in identifying the clinical phenotype which could aid in precise genetic testing. 36 37

In the course of disease progression, upper limb weakness was noted in about two-thirds of patients by 3 years of symptom onset and had limb-girdle weakness as was seen in our previous study. 22 However, in one study from north India, only 16% of them had this feature, and this could be due to the varied time of evaluation after disease onset in these patients. 14

The mean duration of follow-up and mean age at last follow-up were 4.5 and 23.7 years, respectively. The majority of our patients continued to be ambulant at a mean of 10 years after the onset of symptoms suggesting a relatively benign course of the illness. In one of the largest studies by Sáenz et al, among 238 LGMDR1 patients, the mean age at becoming wheel chair dependent was 32.2 years, approximately 18 years after the mean age of disease onset. 32 In a study from Italy, Angelini et al have documented loss of ambulation from 10 to 30 years after disease onset, 25 similar to the study from the United Kingdom, where loss of ambulation occurred at 35.2 years, that is, approximately 22.6 years after onset of symptoms. 38 Similar observations were noted in other European reports. 27 32 Hence, further long-term follow-up of our patients is necessary for assessing the future status of disease progression. In one of our patients with sudden death at 21 years of age, it was suspected to be due to myocardial infarction. In the previous studies, though respiratory insufficiency is frequently observed in calpainopathy patients, a few cases of cardiac involvement are also reported. 39

Mutation analysis in our cohort has shown 47 different mutations, and the spectrum is distributed throughout the length of the *CAPN3* gene ( Fig. 2 ). The majority of mutations are of missense type. Even previous studies have described 34 to 60% of mutations of missense type, and exons 10 and 21 were more susceptible than exons 2, 3, 7, and 12. 24 33

The mutational changes in exon 10, that is, c.1343G > A, and c.1319G > A, leading to missense variation and amino acid substitution occurred in 13.8% of our population. This accumulation and gene pooling in our study cohort could be contributed by consanguinity which is more prevalent in southern India. Overall, 550 delA is the commonest among the Caucasian population with founder effect being demonstrated in Mediterranean and southern European region. 40 However, this mutation was not found in our current Indian cohort. Founder mutations seen in the Brazilian population, R110X, and 2362—2363EG4TCATCT was also not identified in our study. 41 Nevertheless, one founder variant (c.2306G > A) in exon 22, previously described in Amish community of northern Indiana of the United States was detected in one of our patients. 4 Another variant previously described in La reunion island of Indian ocean (c.946–2A > G) was also detected in our series. 35

Seven mutations seen in previously described mutational “hotspot” of exon 21, constituted only approximately 6.9% in our study population. 41 As the types of genetic mutations are known to vary from region to region and with ethnic background, the distribution of *CAPN3* mutations in this study did not show any clear hot spots except for mutation accumulation in exon 10 in 12 cases. We also did not observe any mutations in exons 2, 3, 7, and 12. This wide range of mutations observed in our study could also be due to heterogeneous population of patients with a wide range of ethnicity. In another study from a north Indian center, exon 16 was the commonest mutation site, and approximately 75.6% of mutation groups were located around nine exons. 24 This further strengthens the wide variation of genetic mutation from various regions within India.

There were two patients in the current cohort (Pt_10 and Pt_11) carrying two homozygous variations in *CAPN3* at exon4/11 c.529G > T/c.1448C > T. Both had onset of symptoms at 15 and 18 years, respectively, with features of proximal lower limb weakness with one (Pt_11) having muscle pain as additional symptom. Though both were from different families, they belonged to a single community and during the follow-up duration of 6 years each, both were still ambulant. Both these novel mutations, c.529G > T/c.1448C > T in these patients were likely pathogenic based on ACMG classification suggesting their role in the clinical phenotype. Large exonic deletions are uncommon in *CAPN3* but deletions from exons 2 to 8 have been described before. 17 In our study, large deletions were observed in three unrelated patients. Interestingly, all belong to the Patel community which is believed to originate and be more prevalent in Gujarat. The Agarwal founder mutations: c.2051–1G > T and c.2338G > C were the most recurrent ones in our cohort occurring in total eight patients (compound heterozygous: 6 and homozygous: 2), all belonging to the Agarwal community as described before 23 ( Table 4 ; Supplementary Table S1 , available in online version only). There was no direct correlation observed between the type of genetic mutation and course of disease progression, except those with missense mutation had a more benign course of disease progression than those with nonmissense mutations. However, observation about intrafamilial mutation pattern and disease progression could not be done.

**Table 4:** Recurrent *CAPN3* variants in our cohort

Variants	Number of patients	Patient ID	Location	Zygosity
c.2051–1G > T	7 (9.7%)	Pt_52 to 58	Intron18	Heterozygous
c.2338G > C(p.Asp780His)	7 (9.7%)	Pt_52 to 58	Exon 22	Heterozygous
c.802–9G > A	5 (6.9%)	Pt_15 to Pt_19	Intron 5	Homozygous
c.1319G > A(p.Arg440Gln)	5 (6.9%)	Pt_24 to Pt_28	Exon 10	Homozygous
c.1343G > A(p.Arg448His)	5 (6.9%)	Pt_30 to Pt_34	Exon 10	Homozygous
c.1963delC	4 (5.5%)	Pt_45 to 48	Exon 17	Homozygous
c.145C > T(p.Arg49Cys)	2 (2.7%)	Pt_1, Pt_2	Exon 1	Homozygous
c.304C > T(p.Pro102Ser)	2 (2.7%)	Pt_3, Pt_4	Exon 1	Homozygous
c.529G > T(p.Val177Phe) a	2 (2.7%)	Pt_10, Pt_11	Exon 4	Homozygous
c.1448C > T(p.Ala483Val) a	2 (2.7%)	Pt_10, Pt_11	Exon 11	Homozygous
c.1743dupT(p.Glu582T)	2 (2.7%)	Pt_38, Pt_39	Exon 13	Homozygous
c.1782 + 1G > A a	2 (2.7%)	Pt_42, Pt_43	Exon 17	Homozygous

a Novel variants.

The current study is one of the largest series describing the clinical phenotype, disease progression, and mutation patterns in genetically confirmed LGMDR1 and the first from south India. This study also shows the segregation of *CAPN* 3 mutation in first-order relatives of the family ( Supplementary Table S2 , available in online version only). This study also highlights mutation clusters around few exons, aiding in the diagnosis in limited settings.

## Limitations

Major limitations of this study are that although it has identified genetic mutation clusters, a still larger sample size from each community needs to be screened for these mutations to look for founder effect in this region. Studying intrafamilial genetic variability with phenotypic assessment will further help in understanding this disease pattern. No longitudinal follow-up was performed to investigate the disease progression and natural history.

## Conclusion

Hereby, we describe the genetic mutation pattern of *CAPN* 3 gene and disease progression in Indian, predominantly south Indian patients of LGMDR1. Most of the patients develop symptoms in the second decade with proximal lower limb weakness. The predominant findings include abnormal gait with scapula winging. The majority of the patients remained ambulant even after 10 years of symptoms onset, suggesting a benign course of illness. There were 47 different mutations noted in these patients with 19 novel variants. These novel variants identified in *CAPN3* expand the genotype–phenotype correlation associated with LGMDR1 in Indian population. Our study brings into light the usefulness of NGS for identifying novel variants in LGMDR1 and can be used as a stand-alone molecular diagnostic screening tool for all LGMD-related genes. However, additional confirmation might be required for larger deletions/duplications and complex rearrangements.
