# Supplementary material for: Disease Progression and Mutation Pattern in a Large Cohort of LGMD R1/LGMD 2A Patients from India
Source: Glob Med Genet. 2021 Nov 9;9(1):34–41. doi: 10.1055/s-0041-1736567 (PMC8837411; doi:10.1055/s-0041-1736567)
Supplement: Supplementary file 1 — Supplementary Material [file 10-1055-s-0041-1736567-s2100026.pdf]

Supplementary Table S1 Variants in *CAPN3*

New no	Community	Genomic coordinate (GRCh37/hg19)	dbSNP ID	Nucleotide change (NM_000070.3)	Amino acid change	Variant location	Zygosity	Protein domain/ region	Family history	Het. variant segregation in family members	Clinvar	HGMD accession no.	Novel or Reported	Reference	ACMG
Pt_1	Others	chr15:42652148C>T	rs794726871	c.145C>T	p.Arg49Cys	Exon 1	Hom	–	No	Father, mother	VCV000193037	CM076055	Reported	PMID: 19285864	P
Pt_2	Others	chr15:42652148C>T/ chr15:42684835A>G	rs794726871/ rs1595826673	c.145C>T/ c.946–2A>G	p.Arg49Cys/–	Exon 1/ intron 6	C.Het	–/calpain	No	Father, mother, brother	VCV000193037/ VCV000663747	CM076055/ CS052035	Reported	PMID: 30056071/ PMID: 19556129	P
Pt_3	Others	chr15:42652307C>T	–	c.304C>T	p.Pro102Ser	Exon 1	Hom	Calpain	Yes	^	–	CM053149	Reported	PMID: 16141003	LP
Pt_4	Others	chr15:42652307C>T	–	c.304C>T	p.Pro102Ser	Exon 1	Hom	Calpain	No	Father, mother and paternal grandparents	–	CM053149	Reported	PMID: 16141003	LP
Pt_5	Vokkaliga	chr15:42679950G>A	rs863224964	c.499–1 G>A	–	Intron 3	Hom	Calpain	Yes	Brother (Homo), mother	VCV000217157	CS062040	Reported	PMID: 18055493	P
Pt_6	Vokkaliga	chr15:42679950G>A	rs863224964	c.499–1 G>A	–	Intron 3	Hom	Calpain	No	^	VCV000217157	CS062040	Reported	PMID: 18055493	P
Pt_7	Vokkaliga	Chr15:42387752G>A	rs863224964	c.499–1 G>A	–	Intron 3	Hom	Calpain	No	^	VCV000217157	CS062040	Reported	PMID: 18055493	P
Pt_8	Others	chr15:42678485T>C/ chr15:42689065G>A	–/rs1595831427	c.498+2T>C/ c.1183G>A	–/p.Gly395Arg	Intron3/ exon 9	C.Het	Calpain/calpain	No	^	–/VCV000655419	NR/NR	Reported	–	LP
Pt_9	Others	chr15:42680060C>T	rs763719290	c.608C>T	p.Ala203Val	Exon 4	Hom	Calpain	No	^	VCV000289751	NR	Reported	PMID: 30564623	LP
		chr15:42681242A>G	rs779939785	c.749A>G	p.Lys250Arg	Exon 5	Hom	Calpain	No		VCV000289752	NR	Reported	–	
Pt_10	Others	chr15:42679981G>T/ chr15:42693932C>T	–	c.529G>T/ c.1448C>T	p.Val177Phe/ p.Ala483Val	Exon 4/ exon 11	Hom/ Hom	Calpain/domain III	No	Father	– –	NR/NR*	Novel	–	LP/LP
Pt_11	Others	chr15:42679981G>T/ chr15:42693932C>T	–	c.529G>T/ c.1448C>T	p.Val177Phe/ p.Ala483Val	Exon 4/ exon 11	Hom/ Hom	Calpain/domain III	No	^	– –	NR/NR*	Novel	–	LP/LP
Pt_12	Others	chr15: 42681240delinsAGG	–	c.747_ 760delinsAGG	p.Tyr249Ter	Exon 5	Hom	Calpain	No	^	–		Novel	–	P
Pt_13	Others	chr15:42681193delG/ chr15:42693932C>T	rs1332454949/–	c.700del/ c.1448C>T	p.Val235TrpfsTer18/ p.Ala483Val	Exon 5/ exon 11	C.Het	Calpain/domain III	No	Father	– –	– NR*	Novel	–	P/LP
Pt_14	Others	chr15:42681175A>G/ chr15:42698129delinsAT	–	c.682A>G/ c.1788_ 1789delGAinsAT	p.Met228Val/ p.Lys597Ter	Exon 5/ exon 15	C.Het	Calpain/linker	No	^	– –	–	Novel	–	LP/P
Pt_15	Others	chr15:42682142G>A	rs761211705	c.802–9G>A	–	Intron 5	Hom	Calpain	No	^	VCV000280037	CS053453	Reported	PMID: 16141003	LP
Pt_16	Others	chr15:42682142G>A	rs761211705	c.802–9G>A	–	Intron 5	Hom	Calpain	No	^	VCV000280037	CS053453	Reported	PMID: 16141003	LP
Pt_17	Lingayat	chr15:42682142G>A	rs761211705	c.802–9G>A	–	Intron 5	Hom	Calpain	Yes	Brother (Homo), sister (Homo)/father, mother	VCV000280037	CS053453	Reported	PMID: 16141003	LP
Pt_18	Others	chr15:42682142G>A	rs761211705	c.802–9G>A	–	Intron 5	Hom	Calpain	No	Brother	VCV000280037	CS053453	Reported	PMID: 16141003	LP
Pt_19	Others	chr15:42682142G>A/ chr15:42693952C>T	rs761211705/ rs141656719	c.802–9G>A/ c.1468C>T	–/p.Arg490Trp	Intron 5/ exon 11	C.Het	Calpain/domain III	No	^	VCV000280037/ VCV000166790	CS053453/ CM950194	Reported	PMID: 16141003/ PMID: 33335567	LP/P

(*Continued*)

 © 2021. The Author(s).

Supplementary Table S1 (*Continued*)

New no	Community	Genomic coordinate (GRCh37/hg19)	dbSNP ID	Nucleotide change (NM_000070.3)	Amino acid change	Variant location	Zygosity	Protein domain/ region	Family history	Het. variant segregation in family members	Clinvar	HGMD accession no.	Novel or Reported	Reference	ACMG
Pt_20	Lingayat	chr15:42686537delTA/ chr15:42691830delG	–	c.1113_1114del/ c.1334del	p.Asp371GlufsTer12/ p.Gly445GlufsTer18	Exon 8/ exon 10	C.Het	Calpain/domain III	No	^	–/ –		Novel		P/P
Pt_21	Others	chr15:42689053delG	–	c.1171del	p.Val391SerfsTer43	Exon 9	Hom	Calpain	No	^	–		Novel		P
Pt_22	Others	chr15:42689065G > A	rs1595831427	c.1183G > A	p.Gly395Arg	Exon 9	Hom	Calpain	No	Father	VCV000655419	NR	Reported		LP
Pt_23	Others	chr15:42689079A > C	–	c.1193 + 4A > C	–	Intron 9	Hom	Calpain	Yes	^	–	NR[**]	Novel		VUS
Pt_24	Others	chr15:42691815G > A	rs376107921	c.1319G > A	p.Arg440Gln	Exon 10	Hom	Domain III	No	^	VCV000217147	CM041740	Reported	PMID: 18055493	P
Pt_25	Others	chr15:42691815G > A	rs376107921	c.1319G > A	p.Arg440Gln	Exon 10	Hom	Domain III	Yes	^	VCV000217147	CM041740	Reported	PMID: 18055493	P
Pt_26	Others	chr15:42691815G > A	rs376107921	c.1319G > A	p.Arg440Gln	Exon 10	Hom	Domain III	Yes	^	VCV000217147	CM041740	Reported	PMID: 18055493	P
Pt_27	Others	chr15:42691815G > A	rs376107921	c.1319G > A	p.Arg440Gln	Exon 10	Hom	Domain III	No	^	VCV000217147	CM041740	Reported	PMID: 18055493	P
Pt_28	Others	chr15:42691815G > A	rs376107921	c.1319G > A	p.Arg440Gln	Exon10	C.Het	Domain III	No	Father, mother	VCV000217147	CM041740	Reported	PMID: 18055493	P/LP
		chr15:42701995T > G		c.2003T > G	p.Ile668Ser	Exon 18		EF hand 1			–	NR	Novel		
Pt_29	Others	chr15:42691829G > C	rs773827877	c.1333G > C	p.Gly445Arg	Exon 10	Hom	Domain III	No	Father, mother, brother	VCV000596306	CM990310	Reported	PMID: 32342993	P
Pt_30	Others	chr15:42691839G > A	rs863224956	c.1343G > A[**]	p.Arg448His	Exon 10	Hom	Domain III	No	^	VCV000217149	CM990311	Reported	PMID: 32342993	P
Pt_31	Others	chr15:42691839G > A	rs863224956	c.1343G > A[**]	p.Arg448His	Exon 10	Hom	Domain III	Yes	Brother (Homo), father, mother	VCV000217149	CM990311	Reported	PMID: 32342993	P
Pt_32	Others	chr15:42691839G > A	rs863224956	c.1343G > A[**]	p.Arg448His	Exon 10	Hom	Domain III	No	^	VCV000217149	CM990311	Reported	PMID: 32342993	P
Pt_33	Others	chr15:42691839G > A	rs863224956	c.1343G > A[**]	p.Arg448His	Exon 10	Hom	Domain III	No	Father	VCV000217149	CM990311	Reported	PMID: 32342993	P
Pt_34	Others	chr15:42691839G > A	rs863224956	c.1343G > A[**]	p.Arg448His	Exon 10	Hom	Domain III	Yes	^	VCV000217149	CM990311	Reported	PMID: 32342993	P
Pt_35	Others	chr15:42693986C > T	–	c.1502C > T	p.Thr501Ile	Exon 11	Hom	Domain III	No	Father, mother	–	NR	Novel		LP
Pt_36	Others	chr15:42693952C > T	rs141656719	c.1468C > T	p.Arg490Trp	Exon11	C.Het	Domain III	No	Father, mother	VCV000166790	CM950194	Reported	PMID: 18055493	P
		chr15:42702198C > G	–	c.2115 + 5C > G	–	Intron19		EF hand 2			–	NR	Reported	PMID: 33852849	VUS
Pt_37	Vokkaliga	chr15:42695077G > A	rs398123143	c.1622G > A	p.Arg541Gln	Exon 13	Hom	Domain III	No	^	VCV000092407	CM990318	Reported	PMID: 32668095	LP
Pt_38	Lingayat	chr15:42695198dupT	–	c.1743dupT	p.Glu582Ter	Exon 13	Hom	Domain III	No	^	–	NR	Reported	PMID: 10330340	P
Pt_39	Others	chr15: 42695198dupT	–	c.1743dupT	p.Glu582Ter	Exon 13	Hom	Domain III	No	Father, mother	–	NR	Reported	PMID: 10330340	P
Pt_40	Others	chr15:42695202T > C	–	c.1745 + 2T > C	–	Intron13	Hom	Domain III	No	^	–	NR	Novel		P
Pt_41	Vokkaliga	chr15:42695953_ 42695954insA	–	c.1759dup	p.Thr587AsnfsTer45	Exon 14	Hom	Linker	Yes	Affected paternal cousin (Homo)/ father, mother, paternal uncle, aunt, cousin, maternal aunt	–		Novel		P
Pt_42	Lingayat	chr15:42695976G > A	–	c.1782 + 1G > A	–	Intron14	Hom	Domain III	No	Father, mother	–	NR	Novel		P
Pt_43	Lingayat	chr15:42695976G > A	–	c.1782 + 1G > A	–	Intron14	Hom	Domain III	No	Mother	–	NR	Novel		P

 © 2021. The Author(s).

Supplementary Table S1 (*Continued*)

New no	Community	Genomic coordinate (GRCh37/hg19)	dbSNP ID	Nucleotide change (NM_000070.3)	Amino acid change	Variant location	Zygosity	Protein domain/ region	Family history	Het. variant segregation in family members	Clinvar	HGMD accession no.	Novel or Reported	Reference	ACMG
Pt_44	Lingayat	chr15:42701548delC	rs1566984441	c.1962del	p.Arg655GlyfsTer7	Exon 17	Hom	EF Hand 1	No	Father, mother	VCV000617570		Novel		P
Pt_45	Vokkaliga	chr15:42701548delC	rs1566984441	c.1963delC	p.Arg655GlyfsTer7	Exon 17	Hom	EF hand 1	Yes	Father, mother paternal uncle, aunt, cousin	VCV000617570		Reported		P
Pt_46	Vokkaliga	chr15:42701548delC	rs1566984441	c.1963delC	p.Arg655GlyfsTer7	Exon 17	Hom	EF hand 1	No	^	VCV000617570		Reported		P
Pt_47	Vokkaliga	chr15:42701548delC	rs1566984441	c.1963delC	p.Arg655GlyfsTer7	Exon 17	Hom	EF hand 1	No	^	VCV000617570		Reported		P
Pt_48	Others	chr15:42701548delC	rs1566984441	c.1963delC	p.Arg655GlyfsTer7	Exon 17	Hom	EF hand 1	No	^	VCV000617570		Reported		P
Pt_49	Patel	–	–	Multiple exon del	Multiple exon del	Exon 17–24	Hom	EF hand 1, 2, 3, 4	Yes	^	–				P
Pt_50	Patel	–	–	Multiple exon del	Multiple exon del	Exon 17–24	Hom	EF hand 1, 2, 3, 4	Yes	^	–				P
Pt_51	Patel	–	–	Multiple exon del	Multiple exon del	Exon 17–24	Hom	EF hand 1, 2, 3, 4	No	^	–				P
Pt_52	Agarwal	chr15:42702128G > T/ chr15:42703156G > C	rs886042108	c.2051−1G > T*/ c.2338G > C*	−/p.Asp780His	Intron18/ exon 22	C.Het	Domain III/ EF hand 3	Yes	^	VCV000281184	CS134849	Reported	PMID: 27011640	P
Pt_53	Agarwal	chr15:42702128G > T	rs886042108	c.2051−1G > T*	–	Intron18	Hom	Domain III	No	^	VCV000281184	CS134849	Reported	PMID: 27011640	P
Pt_54	Agarwal	chr15:42702128G > T/ chr15:42703156G > C	rs886042108	c.2051−1G > T*/ c.2338G > C*	−/p.Asp780His	Intron18/ exon 22	C.Het	Domain III/ EF hand 3	No	Father, mother	VCV000281184	CS134849	Reported	PMID: 27011640	P
Pt_55	Agarwal	chr15:42702128G > T/chr15:42703156G > C	rs886042108	c.2051−1G > T*/ c.2338G > C*	−/p.Asp780His	Intron18/ exon 22	C.Het	Domain III/ EF hand 3	Yes	^	VCV000281184	CS134849	Reported	PMID: 27011640	P
Pt_56	Agarwal	chr15:42702128G > T/chr15:42703156G > C	rs886042108	c.2051−1G > T*/ c.2338G > C*	−/p.Asp780His	Intron18/ exon 22	C.Het	Domain III/ EF hand 3	No	^	VCV000281184	CS134849	Reported	PMID: 27011640	P
Pt_57	Others	chr15:42702128G > T/chr15:42703156G > C	rs886042108/ rs778768583	c.2051−1G > T*/ c.2338G > C*	−/p.Asp780His	Intron18/ exon 22	C.Het	Domain III/ EF hand 3	No	Brother^	VCV000281184/ VCV000195641	CS134849/ CM050543	Reported	PMID: 27011640	P/P
Pt_58	Agarwal	chr15:42702128G > T/chr15:42703156G > C	rs886042108/ rs778768583	c.2051−1G > T*/ c.2338G > C*	−/p.Asp780His	Intron18/ exon 22	C.Het	Domain III/ EF hand 3	Yes	^	VCV000281184/ VCV000195641	CS134849/ CM050543	Reported	PMID: 27011640	P/P
Pt_59	Others	chr15:42702170C > T	rs764370512	c.2092C > T	p.Arg698Cys	Exon 19	Hom	EF hand 2	No	^	VCV000285572	CM051885	Reported	PMID: 16650086	LP
Pt_60	Others	chr15:42702794_ 42702795insCAAA/ chr15:42703948C > G	−/rs773318451	c.2192_2193dup/ c.2443C > G	p.His733GlnfsTer4/ p.Leu815Val	Exon 21/ exon 24	C.Het	EF hand 3/ EF hand 4	No	Mother, brother sister	−/−	/NR	Novel		P/LP
Pt_61	Others	chr15:42702813C > T	rs1595847257	c.2212C > T	p.Gln738Ter	Exon 21	Hom	EF hand 3	No	^	VCV000813968	CM053156	Reported	PMID: 16141003	P
Pt_62	Others	chr15:42702844G > A	rs587780290	c. 2243G > A	p.Arg748Gln	Exon 21	Hom	EF hand 3	Yes		VCV000128570	CM970224	Reported	PMID: 9150160	P
Pt_63	Others	chr15:42702844G > A	rs587780290	c. 2243G > A	p.Arg748Gln	Exon 21	Hom	EF hand 3	No	Father, mother	VCV000128570	CM970224	Reported	PMID: 9150160	P
Pt_64	Others	chr15:42702844G > A	rs587780290	c. 2243G > A	p.Arg748Gln	Exon 21	Hom	EF hand 3	Yes	Brother (Homo)	VCV000128570	CM970224	Reported	PMID: 9150160	P
Pt_65	Others	chr15:42703106A > G	rs764459544	c.2288A > G	p.Tyr763Cys	Exon 22	Hom	Domain IV	No	^	VCV000282681	CM994482	Reported	PMID: 15221789	LP
Pt_66	Others	chr15:42703106A > G	rs764459544	c.2288A > G	p.Tyr763Cys	Exon 22	Hom	Domain IV	No	^	VCV000282681	CM994482	Reported	PMID: 15221789	LP

(*Continued*)

 © 2021. The Author(s).

Supplementary Table S1 (*Continued*)

New no	Community	Genomic coordinate (GRCh37/hg19)	dbSNP ID	Nucleotide change (NM_000070.3)	Amino acid change	Variant location	Zygosity	Protein domain/ region	Family history	Het. variant segregation in family members	Clinvar	HGMD accession no.	Novel or Reported	Reference	ACMG
Pt_67	Others	chr15:42703124G > A	rs80338802	c.2306G > A	p.Arg769Gln	Exon 22	Hom	Domain IV	No	Father, mother, brother	VCV000017613	CM950197	Reported	PMID: 7720071	P
Pt_68	Others	chr15:42703127A > G	–	c.2309A > G	p.Tyr770Cys	Exon 22	Hom	Domain IV	No	Father, mother	–	NR	Reported	PMID: 7720071	LP
Pt_69	Others	chr15:42703156G > C	rs778768583	c.2338G > C*	p.Asp780His	Exon 22	Hom	Domain IV	No	Mother, brother, sister	VCV000195641	CM050543	Reported	PMID: 25079074	P
Pt_70	Others	chr15:42703173delTTC	–	c.2355_2357delTTC	p.Phe786del	Exon 22	Hom	Domain IV	No	Mother	–		Reported	PMID: 17596655	LP
Pt_71	Lingayat	chr15:42703203G > A	–	c. 2380 + 5G > A	–	Intron 22	Hom	EF hand 4	No	Father, mother	–	NR	Novel		VUS
Pt_72	Others	chr15:42703948C > G	rs773318451	c.2443C > G	p.Leu815Val	Exon 24	Hom	EF hand 4	No	^	–	NR	Novel		LP

Abbreviations: ^, variant segregation analysis not done in affected family members; ACMG, American College of Medical Genetics and Genomics; B, benign; C.Het, compound heterozygous; CADD, combined annotation dependent depletion; CIP, conflicting interpretations of pathogenicity; D, damaging; DC, disease causing; Het, heterozygous; HGMD, Human Gene Mutation Database-Public version; Homo, homozygous; LP, likely pathogenic; MT2, MutationTaster2 on NCBI37/Ensembl69 build; NR, not reported, NR*, different amino acid change at the same codon position is reported, NR**, c.1193 + 6T>, A is reported; P, pathogenic; PoD, possibly damaging, PHRED score, GRCh37-v1.6; PP2, PolyPhen2 score-v2.2.2r398, PrD-probably damaging; S, sorting intolerant from tolerant (SIFT) score,-v5.1.1, T, tolerated; VUS, variant of uncertain significance.
Note: Its associated protein domain regions; in silico predictions of pathogenicity; ACMG classification and allele frequencies and conservation score. Protein domains referenced from *http://uniprot.org*.

 © 2021. The Author(s).

Supplementary Table S2 All the novel variants are represented based on NCBI reference sequence NM_000070.3

	Serial	Mutations	ACMG attributes	Classification
Pt_10, Pt_11, Pt_13	1	c.529G > T (p.Val177Phe)	PM1, PM2, PP2, PP3	Likely pathogenic
	2	c.1448C > T(p.Ala483Val)	PM1, PM2, PM5, PP2, PP3	Likely pathogenic
Pt_12	3	c.747_760delinsAGG(p.Tyr249Ter)	PVS1, PM2, PP3	Pathogenic
Pt_13	4	c.703del(p.Val235TrpfsTer18)	PVS1, PM2, PP3	Pathogenic
Pt_14	5	c.682A > G(p.Met228Val)	PM1, PM2, PP2, PP3	Likely pathogenic
	6	c.1788_1789delGAinsAT (p.Lys597Ter)	PVS1, PM2, PP3	Pathogenic
Pt_20	7	c.1113_1114del(p.Asp371GlufsTer12)	PVS1, PM2, PP3	Pathogenic
	8	c.1334del (p.Gly445GlufsTer18)	PVS1, PM2, PP3	Pathogenic
Pt_21	9	c.1171del (p.Val391SerfsTer43)	PVS1, PM2, PP3	Pathogenic
Pt_23	10	c.1193 + 4A > C[a]	PM2	VUS
Pt_28	11	c.2003T > G(p.Ile668Ser)	PM1, PM2, PP2, PP3	Likely pathogenic
Pt_35	12	c.1502C > T(p.Thr501Ile)	PM1, PM2, PP2, PP3	Likely pathogenic
Pt_40	13	c.1745 + 2T > C	PVS1, PM2, PP3	Pathogenic
Pt_41	14	c.1759dup (p.Thr587AsnfsTer45)	PVS1, PM2, PP3	Pathogenic
Pt_42, Pt_43	15	c.1782 + 1G > A	PVS1, PM2, PP3	Pathogenic
Pt_44	16	c.1962del (p.Arg655GlyfsTer7) NM_000070.3	PVS1, PM2, PP5	Pathogenic
Pt_60	17	c.2195_2198dup (p.His733GlnfsTer4)	PVS1, PM2, PP3	Pathogenic
Pt_71	18	c.2380 + 5G > A[a]	PM2	VUS
Pt_72	19	c.2443C > G(p.Leu815Val)	PM1, PM2, PP2, PP3	Likely pathogenic

Abbreviations: ACMG, American College of Medical Genetics and Genomics; NCBI, National Center for Biotechnology Information; NM, RefSeq category prefix; Pt, patient. Pathogenic: PVS1, very strong; PM2, moderate; PP3, supporting; PVS1, very strong; PM2, moderate; PP5, supporting. Likely pathogenic: PM1, moderate; PM2, moderate; PP2, supporting; PP3, supporting; PM1, moderate; PM2, moderate; PM5, moderate; PP2, supporting; PP3 moderate; variant of uncertain significance (VUS): PM2, moderate.

[a]Analysis by human splicing finder (*https://hsf.genomnis.com/home*) showed "Alteration of wild type donor site, most probably affecting normal splicing" for both novel intronic variants identified in patients 23 and 71.

 © 2021. The Author(s).
